# Clinically accessible drug-based nano-assemblies with self-targeting ability for NIR-II fluorescence imaging-guided surgery in triple-negative breast cancer

**DOI:** 10.7150/thno.133735

**Published:** 2026-07-22

**Authors:** Ruiqin Yang, Kangliang Lou, Shuangyan Bao, Chen Yang, Yating Gai, Shuanglong Chen, Qingmo Yang, Siqi Qiu, Yilong Lin, Hairong Zhao, Yang Li

**Affiliations:** 1 Department of Breast Surgery, The First Affiliated Hospital of Xiamen University, School of Medicine, Xiamen University, Xiamen 361003, China.; 2 State Key Laboratory of Structural Chemistry & CAS Key Laboratory of Design and Assembly of Functional Nanostructures, Fujian Institute of Research on the Structure of Matter, Chinese Academy of Sciences, Fuzhou 350108, China.; 3 Department of Breast Surgery, The First Affiliated Hospital, Zhejiang University School of Medicine, Hangzhou 310003, China.; 4 Yunnan Provincial Key Laboratory of Entomological Biopharmaceutical R&D, Dali University, Dali 671003, China.; 5 Xiamen Institute for Food and Drug Control, Xiamen 361012, China.; 6 Diagnosis and Treatment Center of Breast Diseases, Shantou Central Hospital, Shantou 515031, China.; 7 Key Laboratory of Biological Nanotechnology NHC. No. 87 Xiangya Road, Changsha, Hunan 410008, China.

**Keywords:** NIR-II fluorescence imaging-guided surgery, pure-drug nano-assemblies, surgical margin, FRα-targeted imaging, breast cancer

## Abstract

**Rationale:**

Accurate intraoperative visualization is critical for reducing margin positivity during breast-conserving surgery for triple-negative breast cancer (TNBC). Second near-infrared (NIR-II) fluorescence imaging represents a promising approach for precision surgery by combining lesion detection with real-time guidance. Nevertheless, the clinical translation of most fluorescence agents remains hampered by carrier-related toxicity and complex synthesis. Therefore, a “green” drug-repurposing strategy was adopted here to construct carrier-free pure-drug nano-assemblies (PDNAs), aiming to provide a biocompatible and precise intraoperative navigation tool for TNBC resection.

**Methods:**

We developed a novel PDNA system (CF-ICG) formed by the simple self-assembly of two clinically employed drugs: calcium folinate and indocyanine green. The targeting specificity of CF-ICG and the feasibility of NIR-II fluorescence-guided surgery were validated using MDA-MB-231-Luc xenograft and MMTV-PyVT transgenic models. A rapid *ex vivo* incubation protocol was developed to differentiate breast cancer from para-cancer tissues.

**Results:**

Driven by intrinsic Ca^2+^ from CF, CF-ICG was assembled through π-π stacking and electrostatic interactions, demonstrating stable physicochemical properties and FRα self-targeting ability. *In vivo* imaging provided high-contrast NIR-II signals for real-time surgical navigation and enabled precise identification of residual submillimeter tumor lesions (diameter ~0.9 mm) in MDA-MB-231-Luc xenograft models. It also clearly differentiated malignant from normal breast tissues in MMTV-PyVT transgenic mice (AUC = 0.941). Furthermore, the diagnostic performance of the rapid *ex vivo* incubation protocol was preliminarily validated using surgical specimens from TNBC patients (n = 11). Notably, this approach effectively differentiated tumors from para-cancer tissues within 12 min (AUC = 0.926).

**Conclusions:**

By combining a “green” fabrication process with a drug-repurposing strategy, we developed CF-ICG as a carrier-free PDNA with tumor self-targeting capability, enabling precise intraoperative navigation in preclinical models and *ex vivo* tissues. These findings support the further development of this approach for more accurate tumor visualization and surgical decision-making in TNBC.

## Background

Breast-conserving surgery (BCS) is currently a standard surgical option for early-stage triple-negative breast cancer (TNBC) patients 1, where securing a negative surgical margin is paramount to reducing local recurrence 2. Nevertheless, solely relying on subjective intraoperative assessments often compromises surgical precision, resulting in high positive margin rates of 15%-50% 3, 4. Although frozen section analysis and other intraoperative diagnostic methods have been applied in clinical practice, they are limited by complicated workflows and long turnaround times 5, 6. Therefore, it is critical to develop rapid and high-accuracy technologies for intraoperative margin assessment.

With high sensitivity, superior spatiotemporal resolution and instant visualization, fluorescence imaging has been widely explored for surgical navigation. Unlike conventional anatomical imaging, it can provide microstructural information at the molecular level 7-9. Indocyanine green (ICG), a clinically approved fluorophore, has been used in fluorescence-guided surgical procedures for several types of cancer 10-13. In addition, ICG shows tail emission in the second near-infrared region (NIR-II, 1000-1700 nm) 14, 15 and has demonstrated potential for intraoperative evaluation of tumor margins 16, 17. However, when used alone, ICG lacks the ability to actively target tumors, which limits its imaging specificity 18. Therefore, introducing targeting moieties that recognize tumor-associated surface biomarkers may help improve its tumor selectivity.

Folate receptor alpha (FRα) is rarely expressed in most normal tissues but is frequently upregulated in multiple solid tumors, including TNBC 19. This expression pattern makes FRα an attractive molecular target for tumor-specific imaging and promotes the development of FRα-targeted probes 20, 21. Among these agents, the folate-based fluorescent probe EC17 has been assessed in clinical trials for margin evaluation during BCS 22. However, the clinical translation of many molecular probes is still limited by strict regulatory requirements and high manufacturing costs. Conventional nanoprobes usually involve complex, multi-step synthetic routes, which increase the difficulty and cost of production. In addition, carrier-based preparations often require comprehensive long-term toxicity assessment, which further prolongs clinical development and raises the risk of translational failure.

Carrier-free pure drug nano-assemblies (PDNAs) have recently been explored as a simple strategy for building nanoscale therapeutic or imaging systems 23, 24. The formation of these nano-assemblies is mainly driven by non-covalent intermolecular forces, including hydrophobic effects, π-π stacking, hydrogen bonds and electrostatic interactions. Compared to traditional nanoprobes, PDNAs offer simplified fabrication, lower production costs, and favorable biocompatibility 25-27. ICG is particularly suitable for this type of assembly because of its amphiphilic structure, which contains hydrophilic sulfonate groups and hydrophobic indole rings. This structure enables ICG to co-assemble with compatible molecules, such as methotrexate 28. In addition, coordination interactions mediated by metal ions (e.g., Cu^2+^, Fe^3+^, Ca^2+^) have been used to facilitate the controlled formation of PDNAs 29.

Herein, we developed a spontaneous Ca^2+^-driven PDNA (CF-ICG) by simply co-assembling two clinically utilized intravenous injections: ICG (for NIR-II imaging) and calcium folinate (CF, serving as the Ca^2+^ source and FRα-targeting ligand). The entire preparation process is rapid and “green”, requiring only simple physical stimulation in a sterile water environment without organic solvents or additional non-FDA-approved components. Based on inherent FRα-targeting of CF, CF-ICG enabled accurate intraoperative identification of residual tumor lesions under NIR-II fluorescence imaging, thereby facilitating a more complete surgical resection. By bypassing the synthetic complexities and carrier-related toxicities of conventional probes, this carrier-free self-assembly strategy provides a promising optical guidance tool, paving a potential route for precise breast cancer surgery.

## Materials and Methods

### Preparation of the CF-ICG probe

Clinically employed CF injection and ICG injection were mixed under continuous shaking to facilitate Ca^2+^-driven self-assembly between the two components. Specifically, 400 μL of CF injection (10 mg/mL) and 400 μL of ICG injection (5 mg/mL) were introduced into 2 mL of sterile water for injection (SWFI). The mixture was continuously vortexed for 12 min to obtain the PDNAs (CF-ICG). To remove unassembled free CF and free ICG molecules, the CF-ICG suspension was purified via dialysis against SWFI using a membrane with a 2000 Da MWCO. Dialysis was performed at 4 ℃ in the dark for 24 h, with the dialysis medium replaced with SWFI every 6 h. Following purification, the CF-ICG suspension was collected for subsequent experiments.

### Characterization of CF-ICG

The absorption and emission spectra of CF-ICG in SWFI (with the ICG concentration equivalent to 50 μg/mL) were measured using a Varioskan Flash multimodal microplate reader and a FLS980 spectrometer (Edinburgh Instruments), respectively. Elemental mapping and compositional analyses were performed via high-resolution transmission electron microscope coupled with energy-dispersive X-ray spectroscopy (FEI Tecnai F20). JEM-1400 transmission electron microscopy (TEM) was employed to visualize the detailed morphology of CF-ICG. The mean hydrodynamic diameter (D*_h_*) and zeta potential were characterized utilizing a Horiba nanoPartica SZ-100 nanoparticle analyzer.

Moreover, to evaluate the stability of CF-ICG within different physiological environments, the samples were incubated in various media, including PBS, 10% FBS, SWFI, and human plasma (purchased from Sigma-Aldrich), and stored in the dark at 25 ℃. The D*_h_* and zeta potential of the CF-ICG in the corresponding solutions were measured at 0, 24, 48, and 72 h. Concurrently, TEM images of CF-ICG were acquired after storage in SWFI at identical intervals. The stability of absorption spectra of the CF-ICG in SWFI was compared with free ICG over the same duration. Furthermore, the fluorescence stability of CF-ICG and ICG was compared in SWFI upon continuous 808 nm laser irradiation (0.1 W/cm^2^).

In order to elucidate the underlying mechanisms by which intermolecular interactions dictate the self-assembly process, we performed competitive binding assays using different chemical agents to induce disassembly: NaCl (a competitive inhibitor for electrostatic interactions), SDS (a competitive inhibitor for hydrophobic interactions), and urea (a competitive inhibitor for hydrogen bonds). The D*_h_* variations of CF-ICG after incubation with SDS, urea, and NaCl were monitored. Quantum chemical calculations and dissipative particle dynamics (DPD) simulations were used to further elucidate the supramolecular interactions during the self-assembly of CF-ICG.

### Cellular uptake study

The targeting capability of CF-ICG toward tumor cells was assessed under the Beckman Coulter CytoFlex S flow cytometer and an upright fluorescence microscope (DM2700 P, Leica). For flow cytometry (FCM), the excitation wavelength was 638 nm and the emission was collected at 780 nm. For upright fluorescence microscopy, fluorescence excitation was provided by a Leica EL6000 external light source, and the emission signal of ICG was collected over the range of 771-829 nm.

The MDA-MB-231 (human-derived) and 4T1 (murine-derived) TNBC cell lines 30, 31 with high FRα expression 32 were used here. Specifically, cells were randomized into Control, CF-ICG, CF-ICG + folic acid (FA). For the CF-ICG group, cells were treated with CF-ICG (with the ICG concentration equivalent to 2 μg/mL) for 6 h. The CF-ICG + FA group were pre-treated with 200 µg/mL FA for 2 h before the addition of CF-ICG. The Control group were treated with an equivalent volume of PBS. Following incubation, cells were rinsed, fixed and subsequently stained with DAPI (to label nuclei, blue pseudocolor) and DiI (to label membranes, red pseudocolor). Cells were then imaged using an upright fluorescence microscope (with green pseudocolor for the ICG channel). To further quantify the targeted cellular uptake, cells from each group were collected and analyzed via FCM.

To comprehensively evaluate the cellular uptake characteristics of CF-ICG, cells were incubated with CF-ICG for different durations (2, 4 and 6 h) or at different equivalent ICG concentrations (0, 0.5, 1, 2 and 4 μg/mL), and the cellular fluorescence intensity was then compared. In addition, CF-ICG uptake in FRα-high MDA-MB-231 cells was compared with ICG uptake in MDA-MB-231 cells and CF-ICG uptake in MCF-10A cells with low FRα expression 32.

### *In vivo* NIR-II fluorescence imaging of subcutaneous tumor-bearing mice

NIR-II imaging were performed utilizing a Series III 900/1700 NIR-II fluorescence imaging system (Suzhou Yingrui Optical Technology Co., Ltd.) under 808 nm excitation, with images collected through a 1000 nm long-pass (LP) filter. Subcutaneous tumor-bearing mice were randomized into three groups (n = 4 per group) and intravenously injected with CF-ICG (2 mg/kg ICG equivalent), free ICG (at the same ICG dose), or CF-ICG following 1 h of FA pre-treatment (50 mg/kg). Fluorescence images were acquired at 3, 6, 12, 24, 36, 48, and 72 h post-injection.

### *In vivo* NIR-II fluorescence-guided surgery in various mouse models

Following the intravenous injection of CF-ICG, MMTV-PyVT and wild-type FVB/N mice (n = 3) were euthanized at 36 h. Guided by the real-time NIR-II images, the mammary glands were then surgically excised. Following *ex vivo* NIR-II imaging, the tissue slices were prepared for hematoxylin and eosin (H&E), immunohistochemistry (IHC) staining, and fluorescence scanning under the Odyssey® CLX imaging system (LI-COR, excitation: 785 nm, collection: 812-832 nm). The mean fluorescence intensities (MFIs) of histologically confirmed tumor tissues and normal tissues were utilized to generate the receiver operating characteristic (ROC) curves. The optimal MFI threshold (Youden’s index) was subsequently identified to maximize both sensitivity and specificity by the formula: Youden’s index = sensitivity + specificity - 1 33.

To evaluate the feasibility of identifying micro-lesions, mice (n = 6) bearing multifocal microtumors (20-40 mm^3^) were subjected to bioluminescence imaging using the IVIS Spectrum imaging system (Caliper Life Sciences) and intravenously administered CF-ICG. At 36 h post-injection, these microtumors were resected under NIR-II imaging guidance and collected for subsequent* ex vivo* NIR-II imaging as well as pathological staining.

Moreover, mice bearing a single tumor were intravenously injected with CF-ICG at 36 h before surgery and randomized into two groups: NIR-II fluorescence- or white light-guided resection (n = 5 for each). Tumor resection was carried out under continuous isoflurane anesthesia. Tumors were initially resected under conventional white light visualization. Subsequently, those mice in the fluorescence-guided group received real-time NIR-II fluorescence imaging to identify any residual fluorescence in the tumor bed. Any tissue with detectable NIR-II fluorescence was removed until no further signal was observed. At 15 days post-operation, mice were monitored for recurrence via bioluminescence imaging. Mice were sacrificed once the recurrent tumor volumes reached 1500 mm^3^ or if they displayed moribund behavior accompanied by significant body weight reduction.

### *Ex vivo* CF-ICG incubation within freshly excised human breast cancer specimens

This clinical study enrolled 11 TNBC patients from The First Affiliated Hospital of Xiamen University, and written informed consent was obtained from all participants. The study was strictly approved by the Institutional Review Board of The First Affiliated Hospital of Xiamen University (No. 2025KY311-01). *Ex vivo* incubation and imaging protocols were performed utilizing paired cancer and adjacent para-cancer samples resected from these patients during surgery. Para-cancer tissues were defined as morphologically normal breast tissues harvested at least 1 cm away from the macroscopic margins of the primary tumor mass. Histologically, these para-cancer tissues contained normal mammary ducts and adipose components without obvious tumor infiltration or atypical hyperplasia. To ensure reproducibility, we strictly followed a Standard Operating Procedure (SOP) during the *ex vivo* incubation and imaging process.

All freshly resected clinical specimens were processed within 30 min post-excision to strictly maintain their biological activity. Tissues were trimmed into 2-mm-thick blocks to minimize potential diffusion-related bias caused by excessive tissue thickness. The rapid incubation process (utilizing CF-ICG or free ICG in SWFI, with the ICG concentration equivalent to 100 μg/mL) was performed in a shaker (25 ℃, 60 rpm) for exactly 7 min. Following incubation, a 5-min rinse in PBST (PBS containing 0.1% Tween-20) was performed in a shaker (25 ℃, 120 rpm) to remove tissue debris and the non-specifically bound probes. NIR-II images were then obtained (excitation wavelength: 808 nm, emission collection wavelength range: above 1000 nm using a 1000 nm LP filter). To ensure consistency across highly variable breast tissue specimens, the sampling regions were strictly defined based on the macroscopic outline of the tissue under white light guidance. The imaging parameters (exposure time: 100 ms, excitation power: 0.1 W/cm^2^) were kept constant across all samples.

Following imaging, the tissue slices were prepared for H&E, IHC staining, and fluorescence scanning under the Odyssey® CLX imaging system (excitation: 785 nm, collection: 812-832 nm). The MFIs of histologically confirmed cancer tissues and para-cancer tissues were used to generate the ROC curves. The optimal MFI threshold (Youden’s index) was then determined.

### Use of AI-assisted tools

ChatGPT (OpenAI) was used during manuscript preparation solely for English language polishing and wording refinement to improve grammar and readability in portions of the Introduction and Discussion sections. The AI tool was not used to generate original scientific content, design the study, collect or analyze data, generate images or figures, interpret results, or draw conclusions.

### Statistical analysis

The NIR-II fluorescence intensities were quantified by Image Studio (LI-COR) and PSLViewer. To assess inter-group differences, independent t-tests and one-way or two-way ANOVA were applied. Mouse survival was analyzed using the Kaplan-Meier curves. All data were derived from a minimum of three independent replicates. Significance was defined at *p* < 0.05. Graph generation and curve fitting were conducted using GraphPad Prism.

## Results

### Synthesis and characterization of CF-ICG

CF-ICG was prepared through a simple “green” synthesis method, as illustrated in Figure 1. To optimize the formulation ratio of CF to ICG, we monitored the D*_h_*, polydispersity index (PDI) and ICG encapsulation efficiency of the assemblies at various CF-to-ICG mass ratios. Our results demonstrated that both the D*_h_* and PDI reached a minimum at a CF-to-ICG mass ratio of 2:1, indicating that the nano-assemblies formed a highly uniform and monodisperse population with PDI < 0.2 (Figure S1A and S1B). Meanwhile, at this 2:1 ratio, the nanoparticles exhibited high stability with minimal variation in D*_h_* during 72 h of storage in SWFI (Figure S1C). Additionally, the encapsulation efficiency of ICG reached ~88.75 ± 2.00% under this ratio (Figure S1D). Therefore, the 2:1 CF-to-ICG mass ratio was selected for all subsequent experiments. Routine physicochemical characterization revealed that CF-ICG formed a clear and transparent green solution, and TEM images demonstrated that the nano-assemblies were monodisperse and spherical (Figure 2A). As shown in Figure 2B, CF-ICG displayed prominent elemental signals of Ca, S, C, N, and O, which are the major constituents of CF and ICG. According to the quantitative elemental analysis based on EDS atomic fractions (Ca: 2.67% and S: 1.93%), a molar ratio of approximately 2.77:1 between CF and ICG was determined within the nano-assemblies (Table S1). Meanwhile, dynamic light scattering analysis indicated that CF-ICG possessed an average D*_h_* of 203.5 ± 22.5 nm (Figure 2C). In contrast to the physical mixture of CF and ICG, CF-ICG exhibited an additional peak at 1081 cm^-1^ in Fourier transform infrared (FTIR) spectroscopy, suggesting the newly formed electrostatic interactions (Figure S2). Moreover, absorbance spectroscopy revealed that the absorption peak of CF-ICG (790 nm) exhibited a 10-nm red shift compared to both free ICG and the CF/ICG physical mixture (both at 780 nm) (Figure 2D). Furthermore, CF-ICG also demonstrated the first near-infrared (NIR-I) and NIR-II emission in SWFI (Figure 2E and 2F). Correspondingly, the NIR-I emission peak of CF-ICG (823 nm) was red-shifted by 4 nm relative to that of free ICG (819 nm). With free ICG serving as the standard reference (quantum yield ~2.9%), the relative NIR-I fluorescence quantum yield of CF-ICG was evaluated to be approximately 3.49 ± 0.23% (Figure S3). This slight enhancement suggested that the ICG molecules within the CF-ICG assembly may be appropriately spaced, thereby effectively suppressing aggregation-caused quenching.

To assess the stability of the probe, the D*_h_*, surface charge and morphological evolution of CF-ICG were monitored. The D*_h_* and surface charge remained relatively stable in different media (PBS, 10% FBS, SWFI and human plasma) across the observation period (Figure 2G and 2I). TEM results also showed that the nanostructure maintained an intact and uniform spherical morphology throughout the storage period without noticeable aggregation or disassembly (Figure 2H). In addition, absorption spectral monitoring confirmed that the absorbance of CF-ICG decayed more slowly over time than that of ICG (Figure 2J and 2K). Furthermore, under continuous 808 nm laser irradiation, the fluorescence intensity of free ICG decreased significantly over time, while CF-ICG retained a higher proportion of its initial fluorescence intensity (Figure S4). These results confirmed the favorable physical and optical stability of CF-ICG, supporting its reliable performance in real-time surgical navigation.

To study the role of intermolecular forces in CF-ICG self-assembly, CF-ICG was treated with SDS, urea, or NaCl to disrupt hydrophobic interactions, hydrogen bonding, or electrostatic interactions, respectively. As the concentrations of NaCl and SDS increased, the D*_h_* of CF-ICG gradually increased (Figure 2L), indicating that the disruption of electrostatic or hydrophobic interactions destabilized the structural integrity of the nano-assembly. In contrast, the D*_h_* remained stable in the presence of urea. These results revealed that hydrophobic and electrostatic interactions were the main forces driving CF-ICG self-assembly.

Through quantum chemical calculations and DPD simulations, the mechanism of supramolecular self-assembly was further clarified. As shown in Figure 2M, CF and ICG formed a stable complex mainly through salt bridges, cation-π interactions and π-π stacking, and the calculated binding energy (ΔE) of the complex was -168.14 kcal/mol. Snapshots from DPD simulations revealed that randomly dispersed CF and ICG molecules progressively assembled into compact nano-aggregates during the 200 ns simulation. Moreover, the total potential energy of the system stayed relatively stable, and the number of π-π stacking interactions between CF and ICG gradually increased. At the same time, hydrogen bonding within the CF-ICG complex remained negligible (Figure 2N-P). Furthermore, the solvent-accessible surface area (SASA) of CF-ICG was significantly lower than that of ICG or CF alone (Figure 2Q), indicating that CF-ICG formed a compact and tightly aggregated structure. These simulation results further confirmed that electrostatic interactions and π-π stacking are the primary driving forces for CF-ICG self-assembly.

### FRα-targeted cellular uptake of CF-ICG *in vitro*

The FRα affinity of CF-ICG was first evaluated by incubating MDA-MB-231 cells (high FRα expression) with escalating concentrations of the nano-probe, followed by quantification of cell-associated fluorescence. CF-ICG exhibited a high binding avidity toward cellular FRα, with a calculated dissociation constant (K_d_) of ~87.74 nM (Figure S5). FRα-mediated internalization of CF-ICG was subsequently evaluated. As illustrated in Figure 3A, MDA-MB-231 cells actively internalized CF-ICG into the cytoplasm, which was markedly suppressed upon pre-treatment with an excess of free FA to block FRα, suggesting that cellular internalization of CF-ICG is specifically mediated by FRα targeting. Consistent with this microscopic observation, MFI analysis showed a similar trend (Figure 3B). Quantitative FCM analysis further confirmed that the uptake of CF-ICG by tumor cells was significantly inhibited after FA blocking (Figure 3C). In addition, prolonging the incubation duration (Figure 3D-F) and increasing the probe concentration (Figure 3G) both led to an enhancement in the fluorescence signal within the MDA-MB-231 cell line. A similar time- and concentration- dependent internalization was also displayed in the 4T1 cell line (Figure 3H and 3I). Besides, the CF-ICG uptake was markedly higher in MDA-MB-231 cells than in normal MCF-10A mammary epithelial cells and was substantially higher than ICG uptake in MDA-MB-231 cells (Figure S6). These findings validated that CF-ICG possesses highly efficient FRα-mediated targeting capabilities in breast cancer cells.

The biocompatibility of CF-ICG was evaluated across various cell lines using the CCK-8 assay. As illustrated in Figure 3J, CF-ICG showed negligible cytotoxicity in normal mammary epithelial cells and breast cancer cells, even at a high ICG-equivalent concentration of 16 μg/mL. The cell viability remained above 90% across all tested concentrations, demonstrating the favorable *in vitro* biocompatibility of the nano-assemblies.

### *In vivo* NIR-II fluorescence imaging and biodistribution

To validate the *in vivo* tumor-targeting capability of CF-ICG, CF-ICG or free ICG was intravenously administered to MDA-MB-231-Luc tumor-bearing mice. For the blocking study, excess FA was injected before CF-ICG administration to occupy FRα binding sites. NIR-II imaging revealed that in both the free ICG and FA-blocking groups, prominent fluorescence signals were detected primarily in the liver, with only negligible accumulation observed in the tumor. In contrast, the CF-ICG group exhibited the most robust and persistent fluorescence signal within the tumor, which remained detectable even at 72 h post-injection (Figure 4A and 4B). Furthermore, the signal-to-noise ratio (SNR) in the CF-ICG group at 36 h post-injection demonstrated a significant enhancement over that of the ICG and FA-blocking groups, highlighting the enhanced tumor-specific accumulation of CF-ICG (Figure 4C).

To further evaluate the systemic biodistribution profile of CF-ICG, major organs and tumors were harvested at 72 h post-administration. *Ex vivo* NIR-II imaging demonstrated that the tumors in the CF-ICG group possessed a significantly higher MFI compared to those in the other groups (Figure 4D). To precisely quantify the tissue distribution, inductively coupled plasma mass spectrometry (ICP-MS) was performed, revealing that the tumor accumulation of the nanoprobes reached approximately 9.66 ± 1.15 %ID/g at 36 h post-administration (Figure S7). Combined fluorescence imaging and ICP-MS results further verified predominant probe enrichment in tumors and livers, with only low-level accumulation detected in other major organs (Figure 4E and S7).

### *In vivo* NIR-II fluorescence-guided surgery in the MMTV-PyVT transgenic mouse model

To assess the diagnostic efficacy of CF-ICG, NIR-II imaging was conducted on an MMTV-PyVT transgenic model with spontaneous breast cancer. *In vivo* NIR-II imaging revealed intense fluorescence signals in malignant mammary glands from transgenic mice. In contrast, normal mammary tissues from wild-type mice exhibited only minimal fluorescence (Figure 5A). After resection under NIR-II fluorescence imaging guidance (Figure 5B), the *ex vivo* malignant tissues also demonstrated an approximately 2.9-fold higher MFI compared to normal controls (Figure 5C and 5D). The diagnostic accuracy was further validated by an ROC curve, yielding an area under the curve (AUC) of 0.941 (95% CI: 0.887-0.995; *p* < 0.0001; Figure 5E). Furthermore, quantitative analysis along the line of interest (LOI) in Figure 5F and 5G revealed that regions ii and iv exhibited superior fluorescence intensity compared to regions i and iii. This fluorescence contrast was corroborated by histopathological assessment: H&E and FRα-IHC staining confirmed that the high-fluorescence regions (ii and iv) contained malignant lesions with high FRα expression, whereas the low-fluorescence regions (i and iii) consisted of normal tissues with low FRα expression (Figure 5H and 5I).

### *In vivo* NIR-II fluorescence-guided surgery in MDA-MB-231-Luc tumor-bearing mice

In order to evaluate the feasibility of CF-ICG to visualize tiny malignant foci during surgery, a multifocal microtumor model was established. As illustrated in Figure 6A and 6B, NIR-II fluorescence signals from microtumors demonstrated a high degree of spatial consistency with bioluminescence signals. Among the 37 microtumors identified by bioluminescence imaging, 35 lesions (94.6%) were detected and removed under intraoperative NIR-II fluorescence guidance. The resected tissues with NIR-II signal were confirmed by pathological examination, revealing that the tumor margins could be clearly delineated under real-time surgical navigation (Figure 6C and 6D). Moreover, the smallest detected lesions were less than 2 mm in diameter, indicating that CF-ICG-based NIR-II imaging could effectively identify microtumors and guide precise resection during surgery.

To further evaluate its surgical applicability, NIR-II fluorescence-guided surgery was performed in mice bearing a single tumor to better mimic clinical tumor resection (Movie S1). As shown in Figure 6E, a small residual tumor that was missed under white light, with a diameter of approximately 0.9 mm, was detected by CF-ICG-based NIR-II imaging. This finding was further confirmed by H&E staining. At 15 days post-surgery, local recurrence was evaluated using bioluminescence imaging. In the NIR-II fluorescence-guided group, no tumor recurrence with bioluminescence signals was observed postoperatively (Figure 6F). However, all five mice (5/5, 100%) in the white light-guided group exhibited recurrent tumor bioluminescence signals (Figure S8). Kaplan-Meier survival analysis further revealed that CF-ICG-guided surgery facilitated a more thorough tumor resection and significantly prolonged overall survival relative to the white-light controls (Figure 6G), supporting the potential value of CF-ICG in real-time surgical navigation.

### *Ex vivo* incubation and NIR-II fluorescence imaging of fresh patient-derived breast cancer tissues

To facilitate intraoperative pathological diagnosis and margin assessment, an *ex vivo* incubation protocol using CF-ICG was evaluated on fresh specimens to differentiate tissue properties and delineate malignancy boundaries. Paired breast cancer and adjacent para-cancer tissues harvested from 11 TNBC patients were divided into CF-ICG and free ICG incubation groups.

As demonstrated in Figure 7A and 7B, CF-ICG exhibited a superior ability to discriminate breast cancer tissues from para-cancer tissues, with a significantly elevated cancer-to-para-cancer fluorescence ratio compared to the free ICG group. Meanwhile, cancer tissues in the CF-ICG group exhibited markedly stronger NIR-II fluorescence relative to matched para-cancer counterparts (Figure 7C), yielding an AUC of approximately 0.926 (95% CI: 0.814-1.000; *p* = 0.0007; Figure 7D). In addition, as shown in Figure 7E, the cancer tissue with high FRα expression demonstrated higher fluorescence than the low-FRα para-cancer and the low-FRα cancer tissue. Owing to the intrinsic heterogeneity of breast cancer, rare FRα-low cancer tissues with diminished fluorescence occasionally led to false-negative outcomes.

Furthermore, we analyzed the fluorescence distribution along the LOI within the cancer tissue (Figure 7F). According to Figure 7F-I, the fluorescence signal clearly delineated the boundaries of the cancer, with regions containing malignant cells (ii and iv) exhibiting significantly stronger fluorescence and higher FRα expression compared to normal tissue areas (i and iii), further supporting the feasibility of CF-ICG for targeted imaging of breast cancer.

### Biocompatibility and systemic toxicity assessment of CF-ICG

To comprehensively evaluate the systemic biocompatibility of CF-ICG, safety assessments were performed. The hemolysis assay verified that CF-ICG did not induce erythrocyte rupture, with the hemolysis rate well below the 5% standard threshold, even at high concentrations (Figure 8A and 8B).

For *in vivo* safety evaluation, body weight monitoring showed no obvious difference between the CF-ICG and PBS groups (Figure 8C). Following CF-ICG administration, blood samples and major organs were collected at 1, 7, and 28 days for subsequent analysis. Histopathological examination of major organs revealed no apparent pathological abnormalities, including necrosis, inflammation, or tissue damage, in either the CF-ICG or PBS group (Figure 8D). Hematological and serum biochemical parameters remained within normal physiological ranges (Figure 8E). These results suggested that CF-ICG possesses favorable biocompatibility and a satisfactory safety profile for *in vivo* applications in surgical navigation.

## Discussion

Fluorescence-guided surgery has been increasingly adopted to enhance intraoperative tumor visualization, facilitating tumor detection and reducing the risk of positive margins 34. Herein, we developed a carrier-free PDNA through the spontaneous Ca^2+^-driven self-assembly between ICG, a clinically approved NIR fluorophore, and CF, a clinically available folate derivative. This design integrates the superior NIR-II fluorescence characteristics of ICG with the inherent FRα-targeting capability of CF, providing high-contrast and real-time tumor visualization in TNBC.

Although PDNAs and ICG-based self-assembled probes have advanced rapidly in recent years 38-40, most current formulations still rely on raw bulk drug powders, toxic organic solvents, surfactants, or non-clinical excipients, which may significantly impede their clinical translation 35-37. In addition, some systems depend on multi-step fabrication procedures or synthetic targeting ligands to achieve tumor specificity, increasing structural complexity and raising additional biosafety concerns, which compromises the original design intention of PDNAs 41-43. In contrast, our approach overcomes these limitations by utilizing two commercially available clinical intravenous injections as the sole starting materials. CF-ICG is prepared in SWFI through a simple and “green” physical process, which can avoid carrier-related toxicity and organic solvent residues. In addition, the assembly is mainly driven by π-π stacking and electrostatic interactions, which support the structural stability of CF-ICG and thereby fulfill an essential prerequisite for reliable surgical guidance. Importantly, a key advantage of this system lies in its multifunctional collaborative assembly strategy: CF serves as both a Ca^2+^ assembly driver (self-driven) and an inherent FRα-targeted ligand (self-targeted), while ICG provides stable NIR-II imaging characteristics. Therefore, this assembly strategy avoids additional chemical modification and achieves multifunctional integration, which is rarely realized in conventional PDNAs.

Because FRα is selectively upregulated in a variety of malignant tissues 44, it has become an important molecular target for tumor-specific imaging and treatment, providing a feasible strategy for precision oncology applications 45. The NIR fluorescence probe pafolacianine (OTL38) has been approved by the FDA and can be used to identify ovarian cancer lesions during surgery 46. However, pafolacianine mainly emits in the NIR-I window (700-900 nm), where high autofluorescence, limited penetration depth, and relatively low SNR may affect imaging performance 47. In contrast, CF-ICG enables NIR-II (1000-1700 nm) imaging, which can provide higher imaging resolution, enhanced penetration depth, and reduced background signals 48-50. Additionally, by combining specific FRα targeting with the optical advantages of NIR-II, CF-ICG can visualize tumor lesions with higher accuracy and is particularly suitable for detecting submillimeter lesions, which is crucial for surgical navigation. Therefore, this method achieved accurate identification of malignant foci in the preclinical model (AUC = 0.941) and enabled more complete removal of malignant tissues than traditional white light-guided surgery. In summary, the NIR-II fluorescence characteristics and FRα-targeting ability of CF-ICG support its potential application in precise margin evaluation during FRα-positive breast cancer surgery.

Moreover, the development of efficient and reliable verification methods is crucial for evaluating the clinical translation potential of new imaging agents. Therefore, we established an *ex vivo* CF-ICG incubation method to rapidly assess fresh tissues obtained during breast cancer surgery. According to our results, this method effectively distinguished between breast cancer and adjacent para-cancerous tissues, with an AUC value of 0.926. The whole process takes only 12 min (7 min for incubation and 5 min for rinsing), which is expected to simplify intraoperative margin evaluation and reduce the time burden of traditional techniques such as frozen section pathology. Future research will focus on pharmacokinetics, biodistribution, and the compatibility of CF-ICG with clinical imaging systems to further evaluate its clinical translational potential.

Several limitations of this study should be noted. First, although FRα is overexpressed in many TNBC cases, its heterogeneous expression may lead to false-negative results in tumors with low FRα levels. Future studies may consider multi-target imaging strategies to address this limitation. Second, CF-ICG showed nonspecific retention in the liver and other reticuloendothelial system (RES) organs, indicating that further refinement of surface properties may be required to minimize off-target accumulation. Third, the clinical validation was based on a relatively limited number of human specimens. Larger cohorts and multicenter studies will be required to establish reliable diagnostic thresholds for standardized surgical decision-making. In addition, although biocompatibility was preliminarily evaluated in animal models, the long-term safety of CF-ICG remains to be further investigated. Furthermore, given the reported photothermal and photodynamic effects of ICG, the therapeutic potential of CF-ICG may be explored in future studies.

## Conclusions

In summary, we developed CF-ICG as a carrier-free NIR-II fluorescent probe through a drug-repurposing strategy and a “green” self-assembly process using clinically available components. CF-ICG shows excellent FRα-mediated tumor targeting ability and enables high-contrast tumor margin delineation as well as precise pathological evaluation in preclinical models and patient-derived tissues. This novel strategy provides a preliminary foundation for advancing precision BCS and may contribute to improved surgical outcomes in patients with TNBC.

## Supplementary Material

Supplementary methods, figures and table.

Supplementary video.

## Figures and Tables

**Figure 1:**
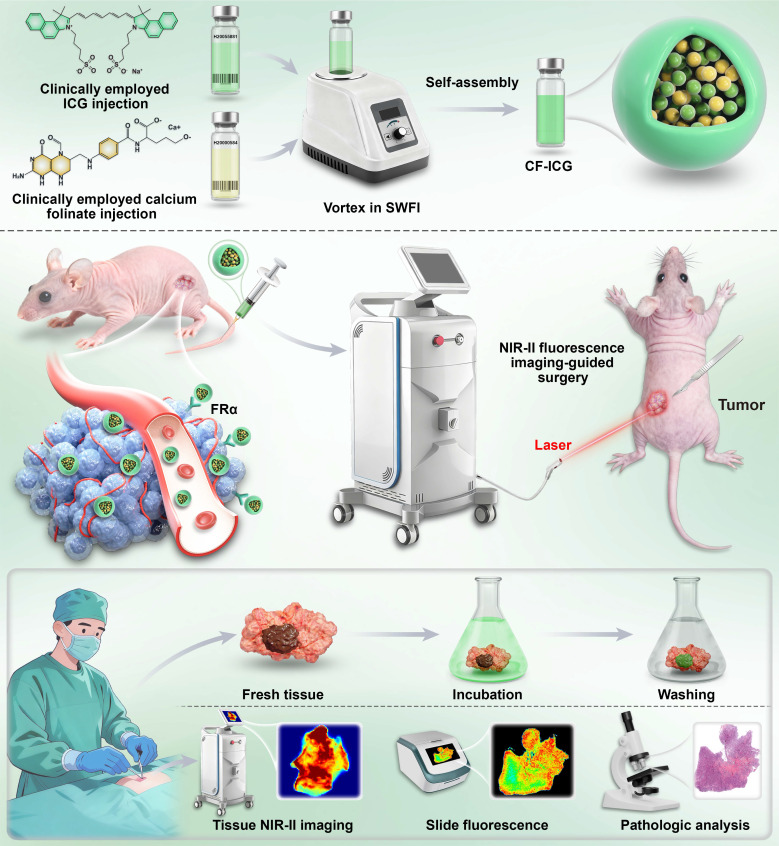
** Schematic illustration of CF-ICG fabrication and its application in NIR-II fluorescence imaging-guided breast cancer surgery.** The top panel illustrates the preparation of CF-ICG via the self-assembly of clinically approved ICG injection and calcium folinate (CF) injection. The middle panel demonstrates the *in vivo* NIR-II imaging-guided evaluation of surgical margins in preclinical models. The bottom panel displays the clinical translation of CF-ICG-based* ex vivo* tissue incubation imaging for the intraoperative evaluation of surgical margins.

**Figure 2:**
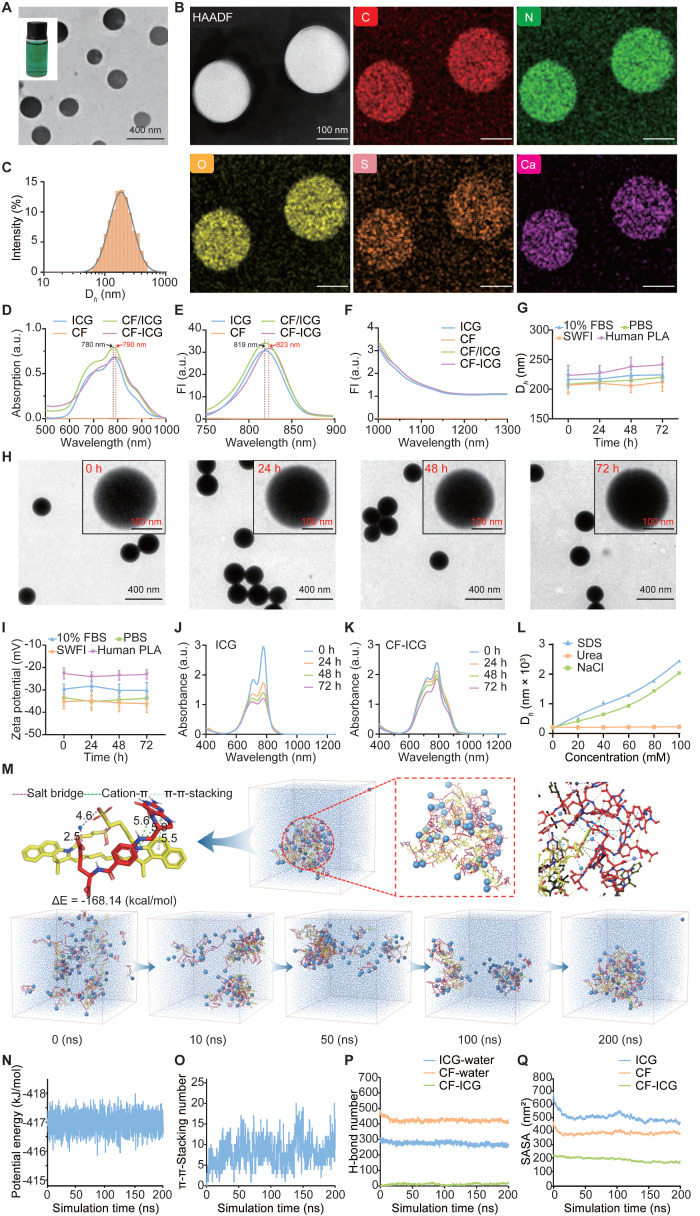
** Characterization of CF-ICG and schematic illustration of its self-assembly mechanism. (A)** Representative transmission electron microscopy (TEM) micrograph of CF-ICG and a digital photograph of the corresponding aqueous solution (Top-left inset). The scale bar represents 400 nm.** (B-C)** EDS element mapping (B) and hydrodynamic diameter (D*_h_*) distribution (C) of CF-ICG. **(D-F)** Absorption (D), NIR-I (E), and NIR-II emission (F) spectra of ICG, CF, the physical mixture of CF and ICG (CF/ICG), and CF-ICG in sterile water for injection (SWFI). Scale bars represent 100 nm in (B).** (G)** Variation of D*_h_* of CF-ICG during storage (0, 24, 48, and 72 h) in different media (PBS, 10% FBS, SWFI, and human plasma). **(H)** Representative TEM micrograph of CF-ICG during storage in SWFI. Scale bars represent 400 nm and 100 nm, respectively. **(I)** Variation of zeta potential of CF-ICG during storage in different media (PBS, 10% FBS, SWFI, and human plasma). **(J-K)** Variations of absorption spectra of ICG (J) and CF-ICG (K) in SWFI during storage. **(L)** D*_h_* variations of CF-ICG after incubation with SDS, urea, and NaCl at different concentrations. **(M)** Self-assembly mechanism of CF-ICG. Quantum chemical calculations revealed salt bridges, cation-π interactions, and π-π stacking between CF and ICG, with a binding energy of ΔE = -168.14 kcal/mol. DPD simulations showed the CF/ICG self-assembly process in water over 0-200 ns (yellow represents ICG, and red represents CF).** (N-Q)** Quantitative analysis of potential energy (N), π-π stacking number (O), hydrogen bond number (P), and solvent-accessible surface area (SASA, Q) during the self-assembly process of CF-ICG.

**Figure 3:**
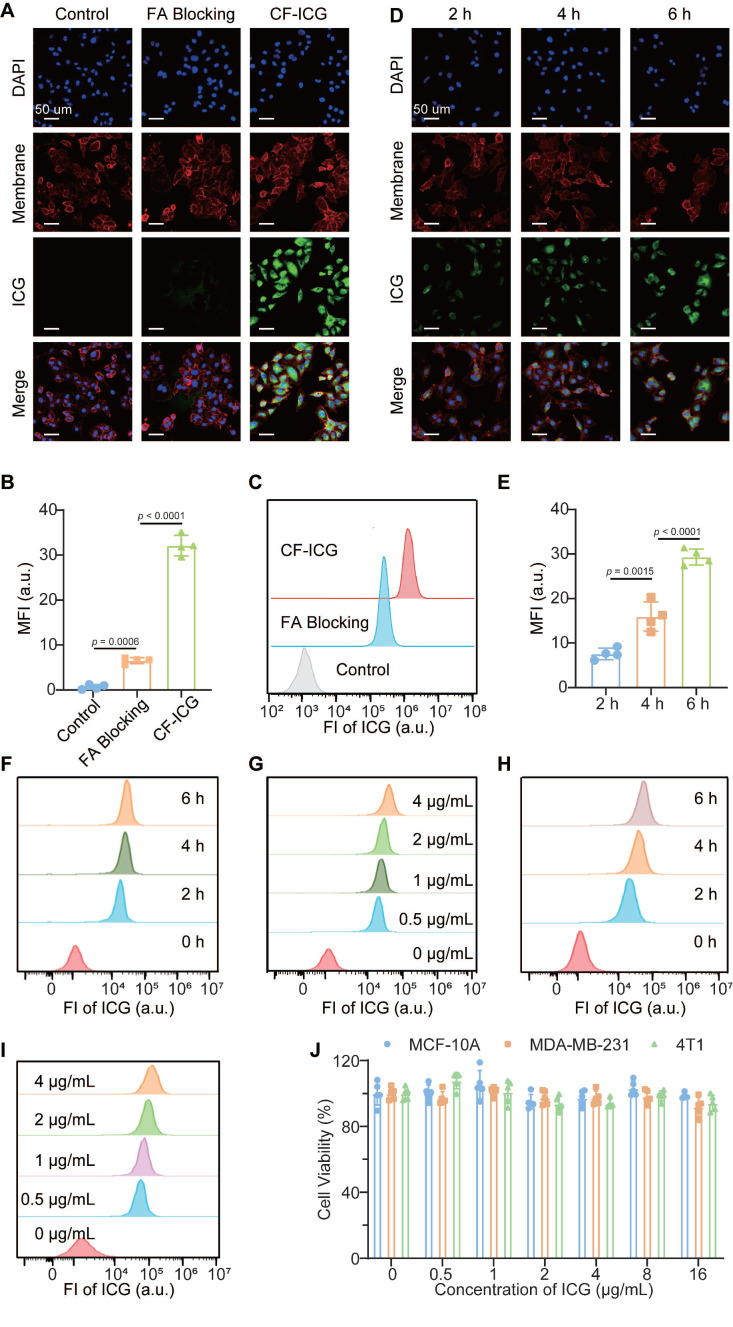
** FRα**-**targeted cellular uptake of CF**-**ICG* in vitro*. (A-B)** Representative upright fluorescence micrographs illustrating the CF-ICG uptake in MDA-MB-231 cells with/without FA blocking (A) and the corresponding mean fluorescence intensity (MFI) analysis (B, n = 4). **(C)** Flow cytometric (FCM) evaluation of the cellular uptake of CF-ICG with/without FA blocking in MDA-MB-231 cells. **(D-E)** Representative micrographs illustrating the CF-ICG uptake in MDA-MB-231 cells at different times (2, 4, and 6 h, D) and the corresponding quantitative fluorescence analysis (E, n = 4). **(F-G)** Cellular uptake evaluation of CF-ICG in MDA-MB-231 cells at different times (2, 4, and 6 h, F) and concentrations (0, 0.5, 1, 2, and 4 μg/mL equivalent ICG, G) via FCM analysis. **(H-I)** Cellular uptake evaluation of CF-ICG in 4T1 cells at various incubation times (H) and concentrations (I) via FCM analysis. **(J)** Cell viability of different cell lines after treatment with different concentrations of CF-ICG (0, 0.5, 1, 2, 4, 8, and 16 μg/mL equivalent ICG) as assessed by the CCK-8 assay (n = 5). All scale bars represent 50 μm. (For FCM, excitation: 638 nm, collection: 780 nm; for upright fluorescence microscopy, excitation was provided by a Leica EL6000 external light source, and the corresponding emission signals were collected at 771-829 nm for ICG, 430-490 nm for DAPI, and 570-590 nm for DiI; mean ± s.d.).

**Figure 4:**
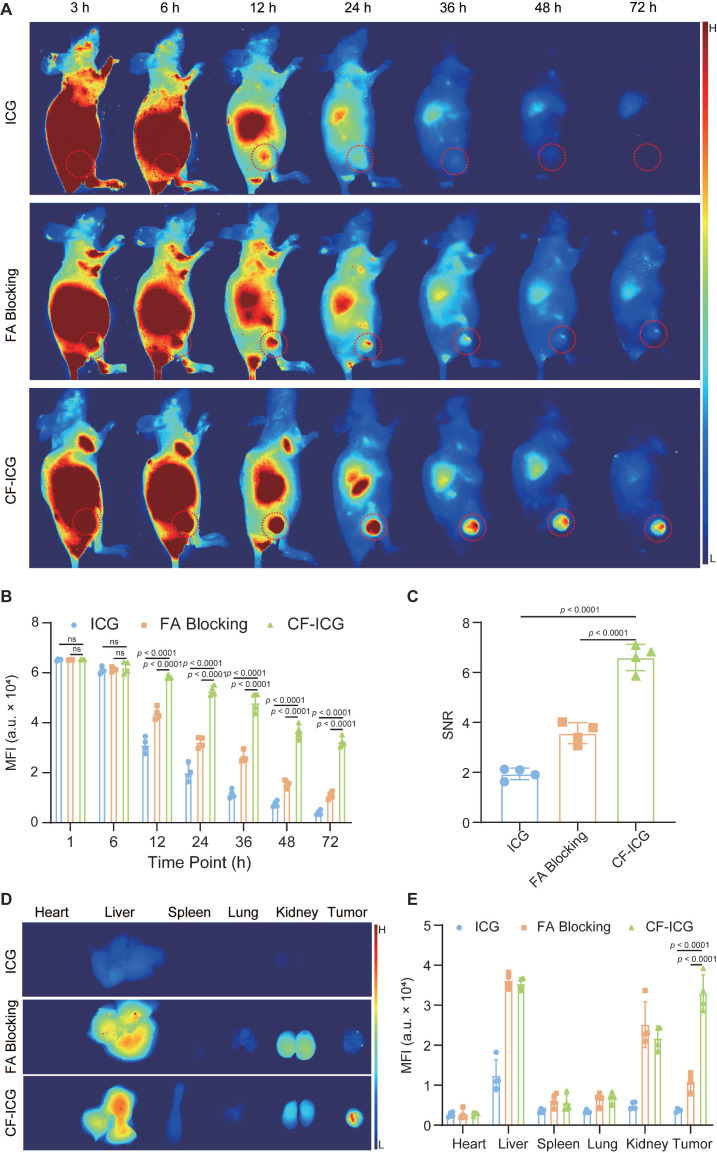
***In vivo* NIR**-**II fluorescence imaging and biodistribution of CF-ICG. (A)** Representative *in vivo* NIR-II images of mice at 3, 6, 12, 24, 36, 48, and 72 h after intravenous administration of ICG, CF-ICG or CF-ICG with excess FA pre-blocking. **(B)** Tumor MFI quantification during NIR-II imaging. **(C)** SNR analysis at 36 h post-injection.** (D-E)** Representative *ex vivo* NIR-II images (D) and corresponding MFI quantification (E) of major organs and tumors at 72 h post-injection. NIR-II images were acquired using a 1000 nm LP filter under 808 nm excitation. (mean ± s.d., n = 4).

**Figure 5:**
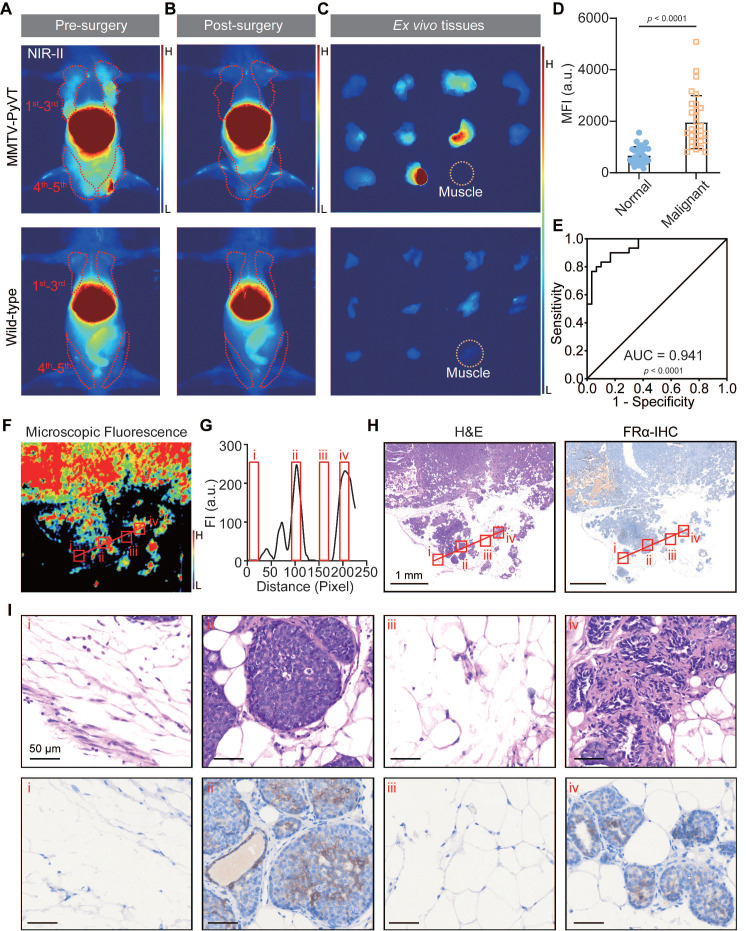
** NIR-II fluorescence**-**guided surgery in the MMTV**-**PyVT transgenic mouse model. (A-C)** Representative *in vivo* NIR-II images of an MMTV-PyVT mouse (top) and a wild-type mouse (bottom) before (A) and after (B) fluorescence-guided surgery, as well as the corresponding *ex vivo* NIR-II images of resected mammary glands (C). The red dotted regions in (A and B) represent the positions of mammary glands, and the yellow dotted regions in (C) represent the muscle tissues. **(D-E)** Quantification of the MFI (D) of *ex vivo* normal and malignant mammary glands and the ROC curve (E) for identifying malignancies. **(F-G)** Representative NIR fluorescence slide scanning result (excitation: 785 nm, collection: 812-832 nm) of the malignant tissue (F) and the corresponding fluorescence intensity (G) along the line of interest (LOI, the red line in F). **(H)** H&E and FRα immunohistochemistry (IHC) results of the tissue from (F). Scale bars represent 1 mm. **(I)** H&E and FRα-IHC results of the tissues marked with red squares (i-iv) along the LOI in (F and H). Scale bars represent 50 μm. NIR-II images were acquired using a 1000 nm LP filter under 808 nm excitation. (mean ± s.d., n = 30).

**Figure 6:**
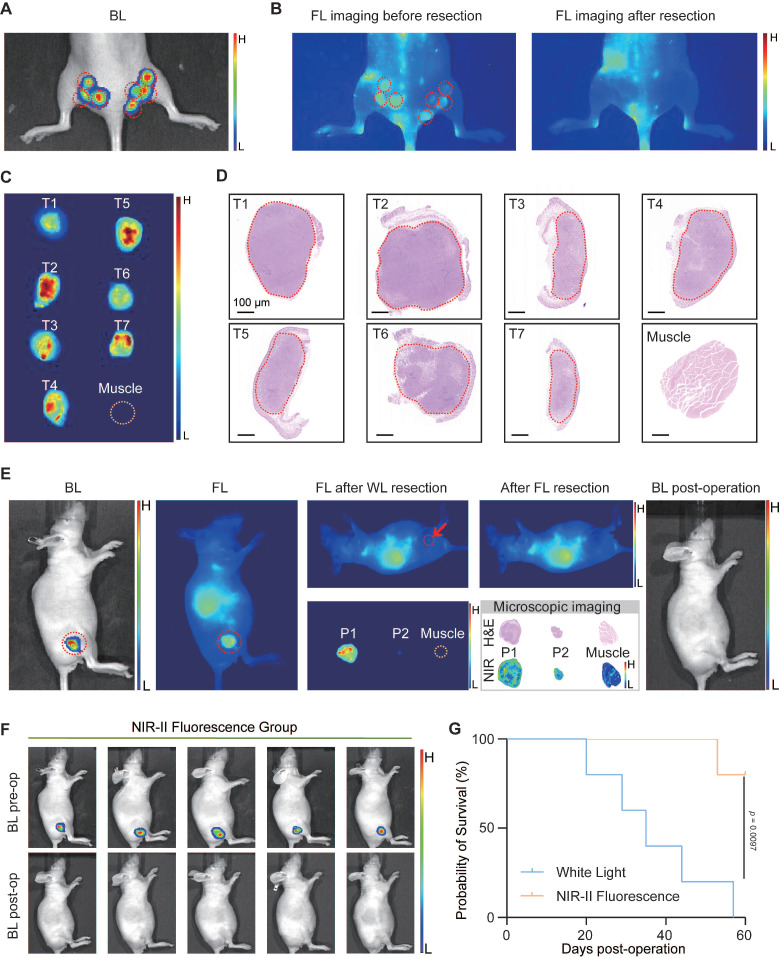
** NIR-II fluorescence**-**guided surgery in MDA**-**MB**-**231**-**Luc tumor-bearing mice. (A-B)** Representative preoperative bioluminescent (BL, A), intraoperative and postoperative* in vivo* NIR-II images (B) of a multifocal microtumor mouse model. The red dotted regions represent the tumors. **(C-D)** *Ex vivo* NIR-II images of resected tumor and muscle tissues (C) and the corresponding H&E-stained histological results (D). The yellow dotted region in (C) represents the muscle tissue, while the red dotted regions in (D) outline the border between the tumor and healthy tissues. Scale bars represent 100 μm. **(E)** Representative preoperative, intraoperative, and postoperative bioluminescent (BL) and NIR-II images of MDA-MB-231-Luc tumor-bearing mice during NIR-II fluorescence-guided surgery, along with the corresponding *ex vivo* NIR-II images, H&E-stained images and NIR fluorescence slide scanning images of resected tissues including the primary tumor (P1), residual tumor (P2) and muscle tissue. The red dotted regions and the red arrow represent the tumors, and the yellow dotted region represents the muscle tissue. NIR fluorescence slide scanning was performed under 785 nm excitation with emission collection at 812-832 nm. **(F)** Preoperative and postoperative BL images of mice undergoing NIR-II fluorescence-guided surgery. **(G)** Kaplan-Meier survival analysis of the NIR-II fluorescence-guided and white light-guided groups. NIR-II images were acquired using a 1000 nm LP filter under 808 nm excitation. (mean ± s.d., n = 5).

**Figure 7:**
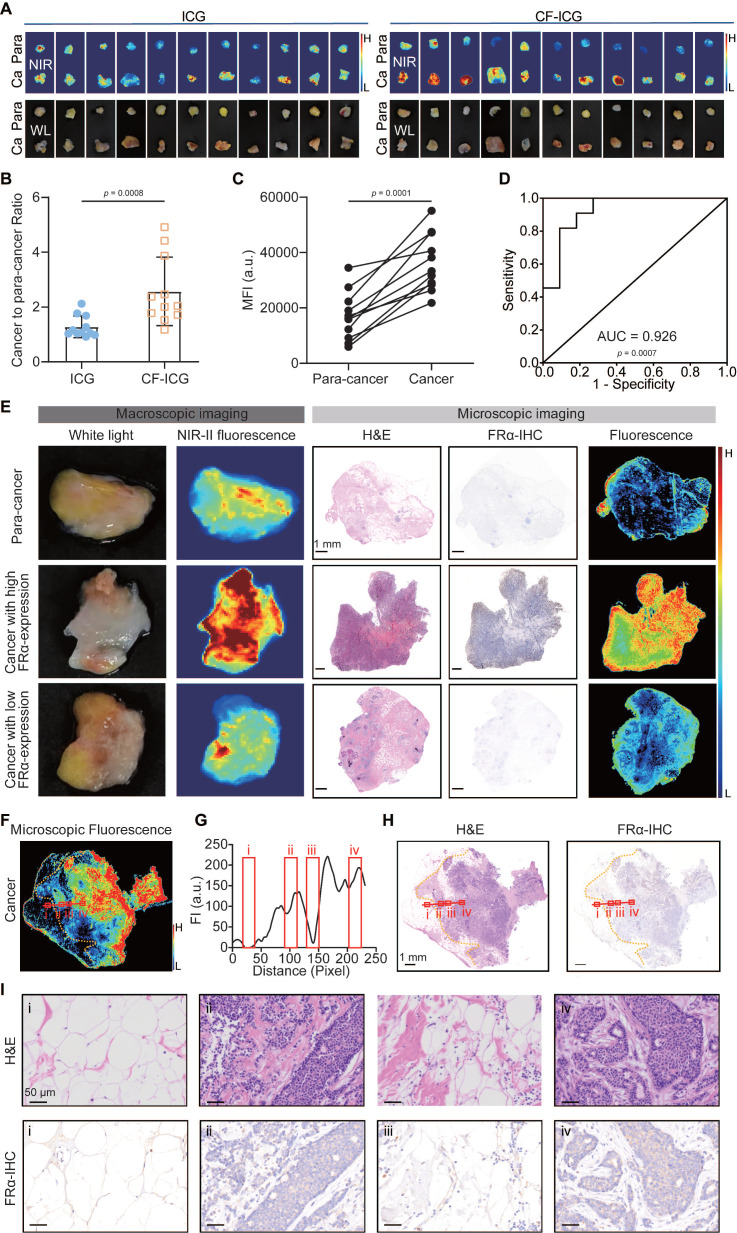
***Ex vivo* incubation and NIR-II fluorescence imaging of fresh patient**-**derived breast cancer tissues. (A-B)** *Ex vivo* NIR-II fluorescence and white-light images of freshly resected cancer and para-cancer tissues after incubation with ICG or CF-ICG (A) and corresponding cancer-to-para-cancer fluorescence ratios (B). **(C-D)** Quantification of the MFI (C) of cancer and para-cancer tissues after incubation with CF-ICG, and the corresponding ROC curve (D) for identifying malignancies. **(E)** Representative white-light and NIR-II fluorescence images of para-cancer, FRα-high and FRα-low cancer tissues after incubation with CF-ICG, along with the H&E staining, FRα-IHC staining and NIR fluorescence slide scanning results. Scale bars represent 1 mm. **(F-G)** Representative NIR fluorescence slide scanning result (excitation: 785 nm, collection: 812-832 nm) of cancer tissue (F) and corresponding fluorescence intensity (G) along the LOI (the red line in F). **(H)** H&E (left) and FRα-IHC (right) results of the tissue from (F). Scale bars represent 1mm. **(I)** H&E (top) and FRα-IHC (bottom) results of the tissues marked with red squares (i-iv) along the LOI in (F and H). Scale bars represent 50 μm. NIR-II images were acquired using a 1000 nm LP filter under 808 nm excitation. (mean ± s.d., n = 11).

**Figure 8:**
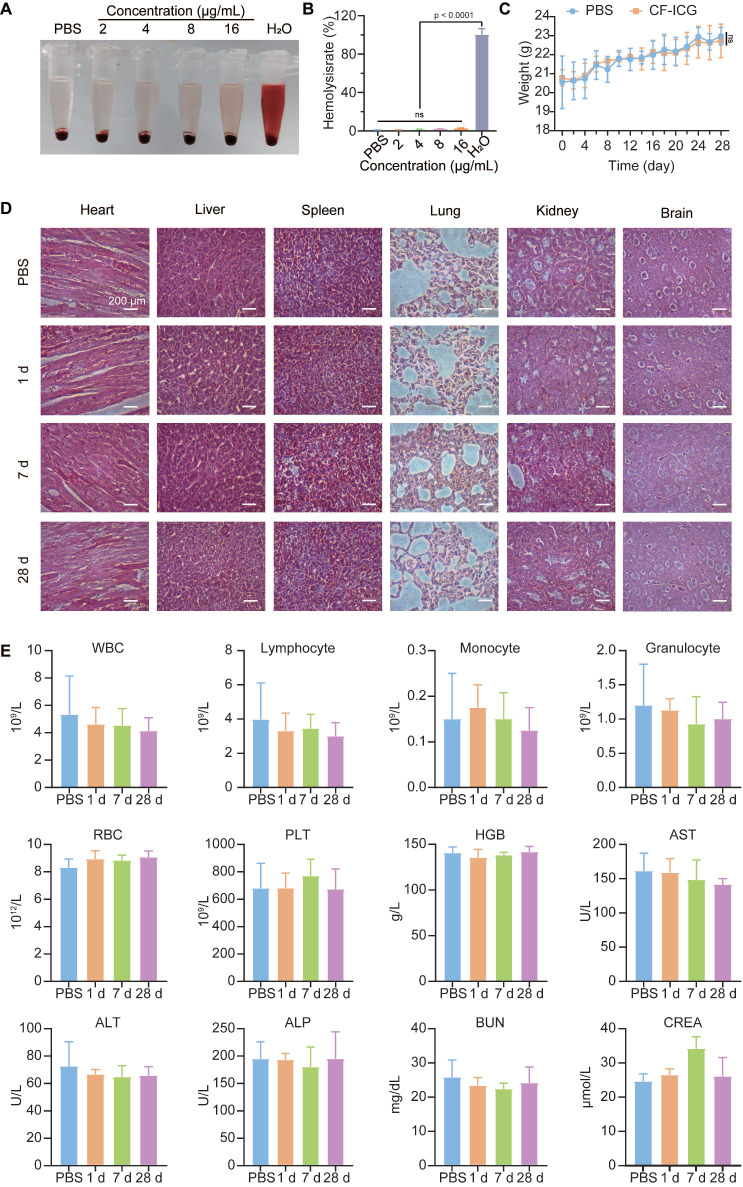
** Biocompatibility and systemic toxicity evaluation of CF**-**ICG. (A-B)** Digital photographs (A) and the corresponding quantification of the hemolysis ratios (B) of red blood cells after incubation with CF-ICG at different concentrations (2, 4, 8, and 16 μg/mL equivalent ICG). **(C)** Changes in body weight of BALB/c mice administered PBS or CF-ICG over a 28-day period. **(D)** Representative H&E staining results of major organs collected from BALB/c mice at 1, 7, and 28 days after CF-ICG or PBS administration. Scale bars represent 200 μm. **(E)** Hematological and serum biochemical parameters of mice at 1, 7, and 28 days after CF-ICG or PBS administration. (n = 4, mean ± s.d.).

## Data Availability

All data that support the findings are included within the paper. Other source data related to this study are available from the corresponding authors upon reasonable request.

## Abbreviations

BCS: breast-conserving surgery
PDNAs: pure-drug nano-assemblies
CF: calcium folinate
NIR-II: second near-infrared
TNBC: triple-negative breast cancer
ICG: indocyanine green
FRα: folate receptor alpha
SNR: signal-to-noise ratio
SWFI: sterile water for injection
MWCO: molecular weight cutoff
ICP-MS: inductively coupled plasma mass spectrometry
DPD: dissipative particle dynamics
FCM: flow cytometry
FA: folic acid
TEM: transmission electron microscopy
D*_h_*: hydrodynamic diameter
SASA: solvent-accessible surface area
MFI: mean fluorescence intensity
LP: long-pass
FTIR: Fourier transform infrared
RES: reticuloendothelial system
ROC: receiver operating characteristic
LOI: line of interest
H&E: hematoxylin and eosin
IHC: immunohistochemistry
AUC: area under the curve
NIR-I: first near-infrared
SD: standard deviation
