# Supplementary material for: Clinically accessible drug-based nano-assemblies with self-targeting ability for NIR-II fluorescence imaging-guided surgery in triple-negative breast cancer
Source: Theranostics. 2026 Jul 22;16(14):8284–301. doi: 10.7150/thno.133735 (PMC13440617; doi:10.7150/thno.133735)
Supplement: Supplementary file 1 [file thnov16p8284s1.pdf]

Supplementary Information

Clinically accessible drug-based nano-assemblies with self-targeting ability for NIR-II fluorescence imaging-guided surgery in triple-negative breast cancer

The supplementary file includes 1 Table and 8 Figures.

Supplementary Methods

Determination of the optimal CF-to-ICG ratio

To optimize the formulation of CF-ICG nanoparticles, different CF-to-ICG mass ratios, including 1:2, 1:1, 2:1, 4:1, and 6:1, were tested during the self-assembly process. For each formulation, the hydrodynamic diameter (D_h), polydispersity index (PDI), D_h stability in sterile water for injection (SWFI), and ICG encapsulation efficiency were measured.

Fluorescence Quantum Yield of CF-ICG

The relative fluorescence quantum yield of CF-ICG ($\Phi_{CF\text{-}ICG}$) in water was determined using free ICG as the standard reference. According to previous reports [1, 2], the NIR-I quantum yield of ICG in water was taken as 2.9%. CF-ICG and ICG were prepared in SWFI at an equivalent ICG concentration of 5 μg/mL, and their NIR-I fluorescence emission spectra were recorded. $\Phi_{CF\text{-}ICG}$ was calculated using the following equation [3]: $\Phi_{CF\text{-}ICG} = \Phi_{ICG} \times (I_{CF\text{-}ICG}/I_{ICG}) \times (A_{ICG}/A_{CF\text{-}ICG}) \times (\eta_{CF\text{-}ICG}^2/\eta_{ICG}^2)$. Φ: Fluorescence quantum yield, *I*: Integrated fluorescence emission peak area, *A*: Absorbance at the excitation wavelength, η: Refractive index of the solvent.

In Vitro Receptor-Binding Affinity Assay

The binding affinity of CF-ICG for FRα was examined using MDA-MB-231 cells with high FRα expression. 1×10^6 cells were resuspended in fresh medium containing different concentrations of CF-ICG (0, 12.5, 25, 50, 100, 200, 400, 800, and 1600 nM, calculated based on ICG concentration). The cell suspensions were incubated at 4 °C for 30 min. The cells were washed and then lysed with 1.0 mL of 1% SDS in saline. Cell-bound fluorescence was quantified using a fluorescence spectrometer. The binding affinity (dissociation constant, K_d) was determined by non-linear regression analysis of the percentage of cell-bound fluorescence against CF-ICG concentration [4].

Animal Preparation

BALB/c-Nude mice (6-8 weeks, female) were purchased from Guangdong Yaokang Biological Technology Co. Ltd. All animal experiments were approved by the Institutional Ethical Committee of Animal Experimentation at Xiamen University (No. XMULAC20220145) and conducted in accordance with the guidelines of the Association for Assessment and Accreditation of Laboratory Animal Care International. The MDA-MB-231-Luc subcutaneous tumor model and MMTV-PyVT transgenic model were established as previously described [5].

In vivo biodistribution evaluation via ICP-MS

Subcutaneous tumor-bearing mice were administered CF-ICG (2 mg/kg, for ICG quantification). At 12, 36, and 72 h after injection, the major organs and tumors were harvested, weighed, and digested in a mixture of HNO_3 and H_2O_2 (HNO_3/H_2O_2 = 2:1 by volume). The biodistribution of CF-ICG was quantified by Inductively Coupled Plasma Mass Spectrometry (ICP-MS) based on Ca^{2+} content.

Supplementary Figures

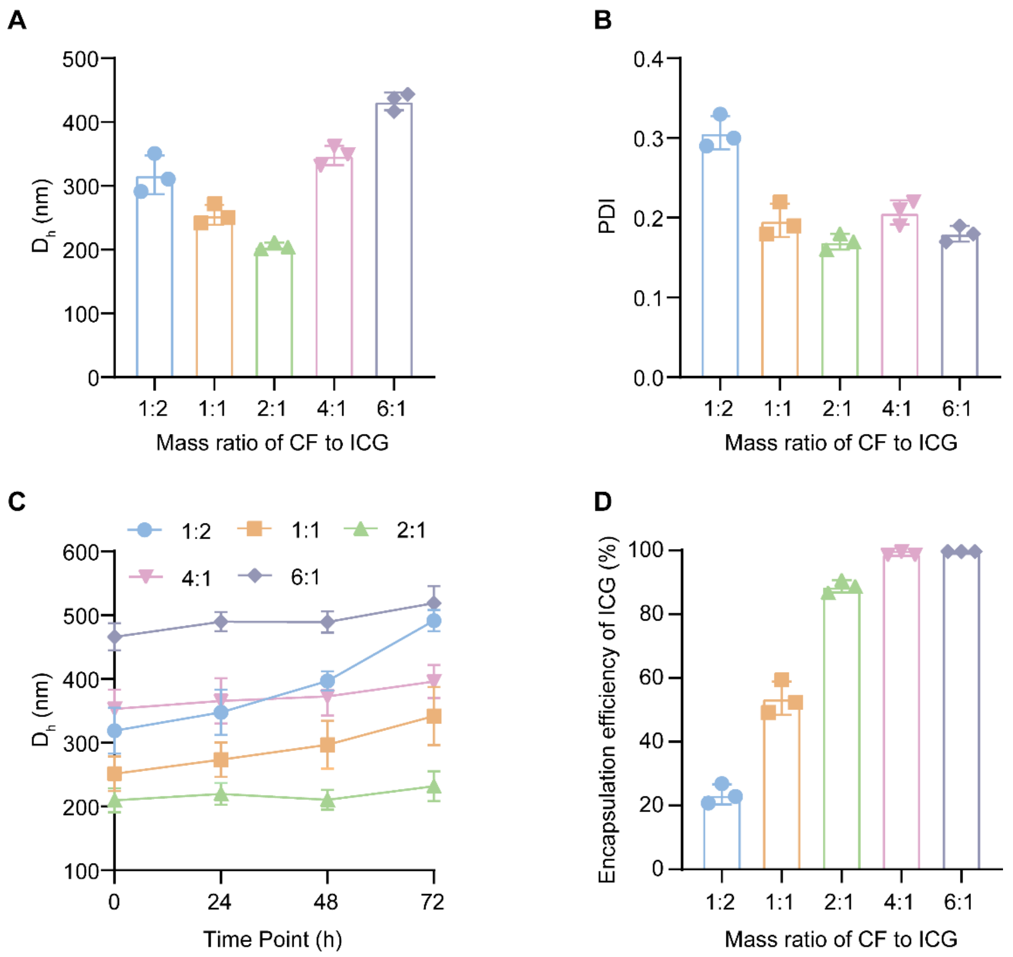

Figure S1 The D*h* (A), polydispersity index (PDI, B), D*h* stability in SWFI (C) and ICG encapsulation efficiency (D) of the assembly with different CF-to-ICG mass ratios.

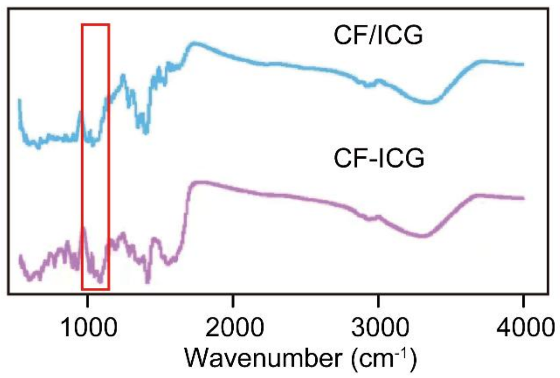

Figure S2 Fourier transform infrared (FTIR) spectra of the physical mixture (CF/ICG) overlapped with the FTIR spectra of CF-ICG.

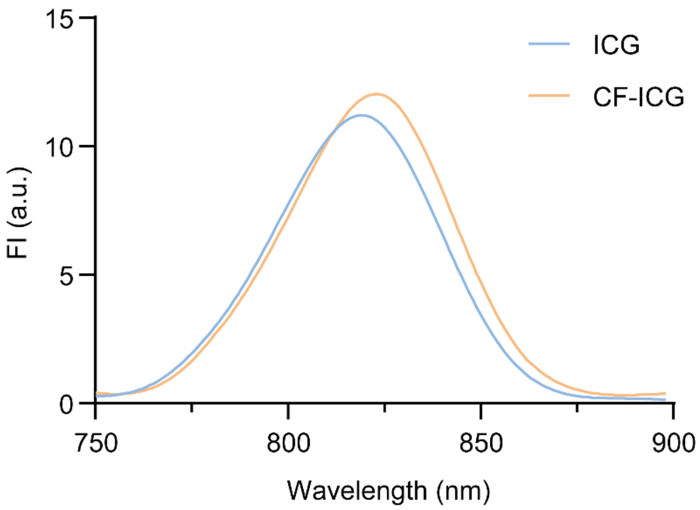

Figure S3 The NIR-I emission spectra of CF-ICG and an equivalent amount of free ICG (equivalent ICG concentration: 5 μg/mL) in SWFI.

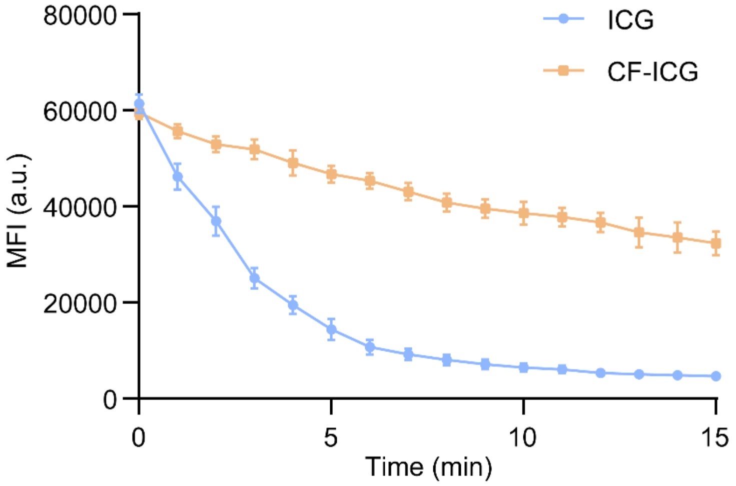

Figure S4 Variation of the MFI of ICG and CF-ICG in SWFI during continuous 808 nm laser irradiation (0.1 W/cm^2).

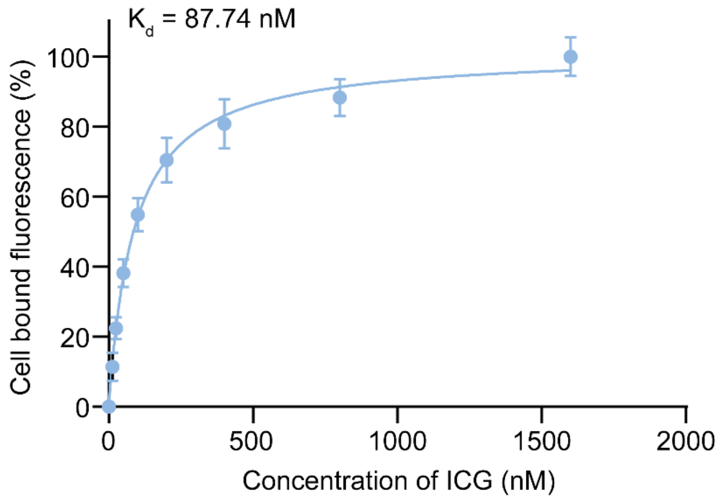

Figure S5 Dose-dependent binding of CF-ICG to MDA-MB-231 cells (high FRα expression, n = 3).

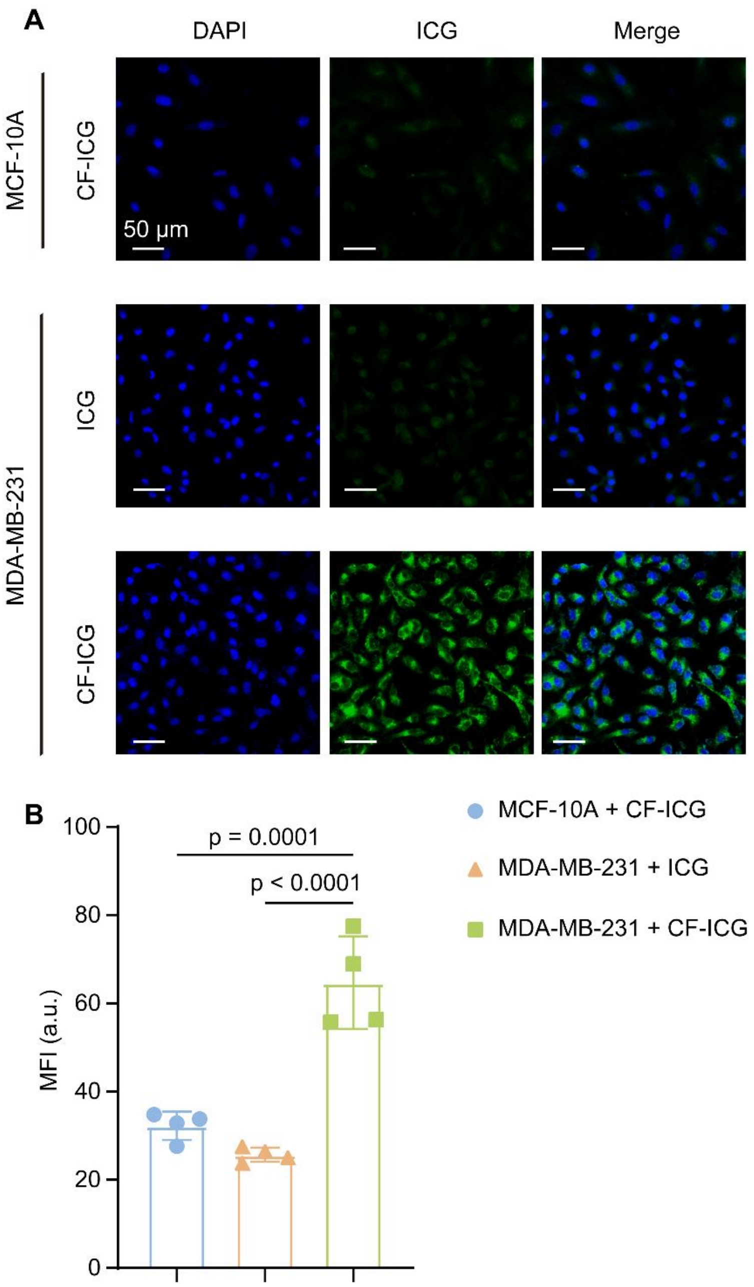

Figure S6 Representative micrographs (A) and MFI (B) illustrating the uptake of CF-ICG by MCF-10A cells and the uptake of ICG or CF-ICG by MDA-MB-231. All scale bars represent 50 μm. (n = 4, mean ± s.d.)

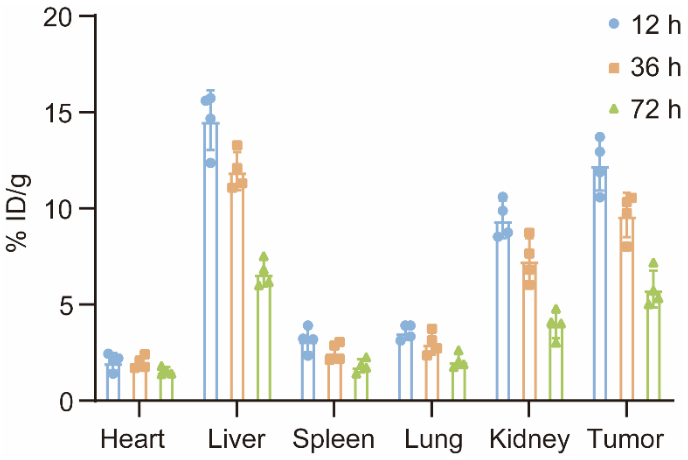

Figure S7 Time-dependent biodistribution of major organs and tumors after intravenous injection of CF-ICG for different periods via ICP-MS. (n = 4, mean ± s.d.)

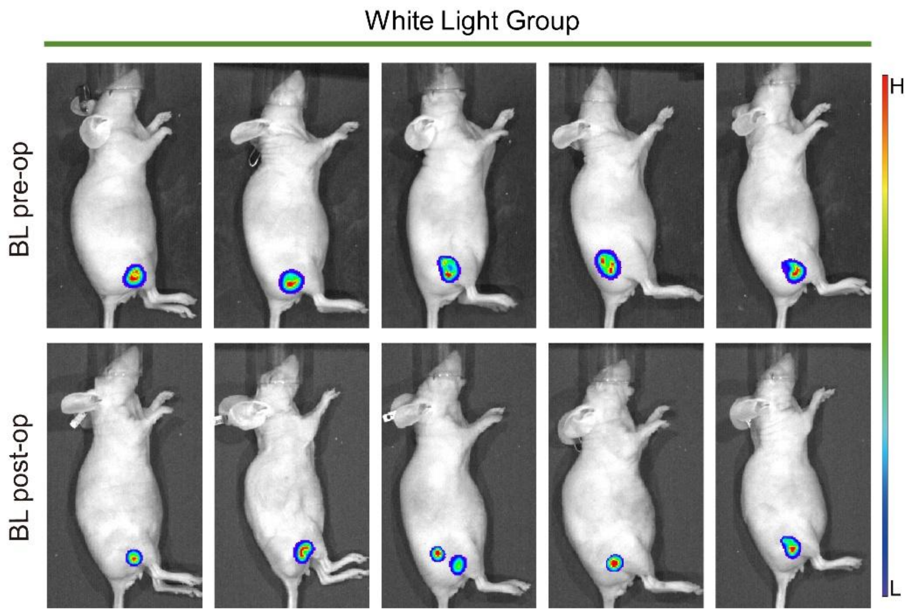

Figure S8 Preoperative and postoperative bioluminescent images of mice undergoing white light-guided surgery.

Table S1. Elemental composition and estimated molar ratio of CF-ICG Nano-assemblies derived from EDS analysis

Element	Atomic Fraction (%)	Associated Molecule	Characteristic Atoms per Molecule	Relative Molar Amount	Calculated Molar Ratio (CF:ICG)
Ca (Calcium)	2.67	CF	1 (Ca)	2.67 / 1 = 2.67	~2.77:1
S (Sulfur)	1.93	ICG	2 (S)	1.93 / 2 = 0.965	

References

1. Cosco ED, Lim I, Sletten EM. Photophysical Properties of Indocyanine Green in the Shortwave Infrared Region. ChemPhotoChem. 2021; 5: 727-34.
2. Gamage RS, Smith BD. Fluorescence Imaging Using Deep-Red Indocyanine Blue, a Complementary Partner for Near-Infrared Indocyanine Green. Chem Biomed Imaging. 2024; 2: 384-97.
3. Rurack K. Fluorescence Quantum Yields: Methods of Determination and Standards. In: Resch-Genger U, editor. Standardization and Quality Assurance in Fluorescence Measurements I: Techniques. Berlin, Heidelberg: Springer Berlin Heidelberg; 2008.
4. Kularatne SA, Thomas M, Myers CH, Gagare P, Kanduluru AK, Crian CJ, et al. Evaluation of Novel Prostate-Specific Membrane Antigen-Targeted Near-Infrared Imaging Agent for Fluorescence-Guided Surgery of Prostate Cancer. Clin Cancer Res. 2019; 25: 177-87.
5. Yang RQ, Chen M, Zhang Q, Gao YY, Lou KL, Lin TT, et al. Development and Preclinical Evaluation of a Near-Infrared Fluorescence Probe Based on Tailored Hepatitis B Core Particles for Imaging-Guided Surgery in Breast Cancer. Int J Nanomedicine. 2022; 17: 1343-60.
